# Prediction of Date Fruit Quality Attributes during Cold Storage Based on Their Electrical Properties Using Artificial Neural Networks Models

**DOI:** 10.3390/foods11111666

**Published:** 2022-06-06

**Authors:** Maged Mohammed, Muhammad Munir, Aljazi Aljabr

**Affiliations:** 1 Date Palm Research Center of Excellence, King Faisal University, Al Hofuf 36362, Saudi Arabia; mmunir@kfu.edu.sa; 2 Department of Agricultural and Biosystems Engineering, Faculty of Agriculture, Menoufia University, Shebin El Koum 32514, Egypt; 3 Date Palm Research Center Al-Ahsa, Ministry of Environment, Water and Agriculture, Al Mubarraz 36321, Saudi Arabia; jazi.jabr@gmail.com

**Keywords:** pH, water activity (a_w_), moisture content (MC), total soluble solids (TSS), Machine Learning (ML), Artificial Neural Networks (ANNs), Multiple Linear Regression (MLR)

## Abstract

Evaluating and predicting date fruit quality during cold storage is critical for ensuring a steady supply of high-quality fruits to meet market demands. The traditional destructive methods take time in the laboratory, and the results are based on one specific parameter being tested. Modern modeling techniques, such as Machine Learning (ML) algorithms, offer unique benefits in nondestructive methods for food safety detection and predicting quality attributes. In addition, the electrical properties of agricultural products provide crucial information about the interior structures of biological tissues and their physicochemical status. Therefore, this study aimed to use an alternative approach to predict physicochemical properties, i.e., the pH, total soluble solids (TSS), water activity (a_w_), and moisture content (MC) of date fruits (Tamar), during cold storage based on their electrical properties using Artificial Neural Networks (ANNs), which is the most popular ML technique. Ten date fruit cultivars were studied to collect data for the targeted parameters at different cold storage times (0, 2, 4, and 6 months) to train and test the ANNs models. The electrical properties of the date fruits were measured using a high-precision LCR (inductance, capacitance, and resistance) meter from 10 Hz to 100 kHz. The ANNs models were compared with a Multiple Linear Regression (MLR) at all testing frequencies of the electrical properties. The MLR models were less accurate than ANNs models in predicting fruit pH and had low performance and weak predictive ability for the TSS, a_w_, and MC at all testing frequencies. The optimal ANNs prediction model consisted of the input layer with 14 neurons, one hidden layer with 15 neurons, and the output layer with 4 neurons, which was determined depending on the measurements of the electrical properties at a 10 kHz testing frequency. This optimal ANNs model was able to predict the pH with R^2^ = 0.938 and RMSE = 0.121, TSS with R^2^ = 0.954 and RMSE = 2.946, a_w_ with R^2^ = 0.876 and RMSE = 0.020, and MC with R^2^ = 0.855 and RMSE = 0.803 b by using the measured electrical properties. The developed ANNs model is a powerful tool for predicting fruit quality attributes after learning from the experimental measurement parameters. It can be suggested to efficiently predict the pH, total soluble solids, water activity, and moisture content of date fruits based on their electrical properties at 10 kHz.

## 1. Introduction

Date palm (*Phoenix dactylifera* L.) is one of the oldest fruit trees that grow widely in the Middle East and North Africa. Dates are a key source of income and a staple food for locals in many regions where they are cultivated. They have also played an essential role in the socioeconomic and environmental conditions of those countries [1]. The demand for dates has increased significantly in date-importing countries like India, Germany, the United Kingdom, the USA, Netherlands, Canada, Spain, Italy, Belgium, and Switzerland. Given the importance of the date palm trade on a local and global scale, ensuring a continuous supply in the market is vital [2]. Therefore, it is imperative to preserve date fruits in cold storage to maintain their supply chain. The fully ripe date palm fruits (Tamar) can be stored at 0–5 °C for 6–12 months [3,4,5,6]. Stored date fruits (cv. Khalas) at freezing temperature for up to 6 months reduced fruit weights, moisture content, pH, titratable acidity ratio, and pectin, while their total soluble sugars and titratable acidity increased [7]. Date palm cvs. Sukkary and Khalas had their fruit size, water activity, pH, and redness color reduced after being stored at 5 °C for 12 months. However, fruit firmness and color (lightness and yellowness) increased [8].

Food security necessitates the storage of fruits in suitable conditions. The primary goals of fruit storage are to preserve fruits for consumption out of season, keep food in good shape, slow down fruit decaying, ensure an even supply to the market, and acquire higher pricing [9,10,11]. The flavor, color, texture, and nutrients of fruits are preserved when correctly stored. The most critical factors that affect the longevity of fruits after harvesting and during storage are temperature and relative humidity [12,13,14]. Optimal relative humidity helps prevent weight loss, the spread of fungal diseases, and physiological disorders [5,15]. Lowering these factors to a suitable level is one approach to slow down the deterioration of fruits and hence increase the fruit preservation time during storage [16,17]. Every fruit has a ‘critical temperature’ below or above where unfavorable and irreversible chemical reactions occur. Therefore, high but not saturated relative humidity is required for most stored fruits [18,19,20,21,22,23].

Many studies on the quality assessment of agricultural products before and after storage have been conducted. Fruits have substantially contributed to these studies due to their widespread production and consumption [24]. Over the last few decades, several scientists have established a variety of methodologies for evaluating the quality of agricultural products other than analytical laboratory techniques [25,26]. The quality of consumable products such as fruits and vegetables comprise multiple characteristics. Sensory properties (appearance, texture, taste, and aroma), nutritional values, physicochemical and mechanical properties, functional properties, and defects are all considered when determining the quality of a product [27]. Nondestructive methods are widely used to measure fruit and vegetable quality; it is precise and rapid, making them ideal for online applications. All production and distribution chain activists, such as insurance companies, packaging, and transportation businesses, wholesalers, and retailers, benefit from nondestructive instrumental research in fruit firmness assessment. These studies can be used to assess fruit quality, predict the best time for their harvest, classify them according to their quality degree, and detect visible and internal fruit defects [26,28,29,30,31,32]. The measurement of electrical properties of biological materials permitted the possibility of a solution to the challenge of nondestructive fruit quality evaluation simply and quickly [33,34]. Changes in electrical characteristics can infer interior quality changes indirectly [35]. Physical properties as indicators for food quality can be used to determine food quality through electric conductivity or resistivity measurements [33,36,37]. The electrical properties of fruits have also been studied, such as the electrical impedance, resistance, and reactance [38,39,40].

In agricultural products, the electrical properties of cell tissues have been studied as indicators for cell tissue integrity. The electrical characteristics of cell membranes are distinct, and when the cell membrane is disrupted, these qualities decline [39,41]. The amount of water in biological cells is critical for their structural and functional integrity [42]. Therefore, structural changes in the cell membrane are supposed to cause a decrease in cell membrane capacitance when fruit moisture is decreased [43]. Extracellular resistance has been found to decrease as cell membrane integrity deteriorates during storage [44]. Electrical impedance spectroscopy is used as a rapid indicator for the freshness of fruit [45]. The impedance and capacitance of the cell membrane drop substantially as the electricity frequency rises [46].

The acquisition of digital data of the physicochemical properties is essential, and real-time quality monitoring is unattainable without it. The conventional procedures are time-consuming and ineffective for this cutting-edge technology. Artificial intelligence models are currently simple and have sparked interest in agriculture. Neurocomputing does not require the formulation of rules or algorithms, which affects a software’s performance [47]. It is easy to collect data and monitor food quality with the Internet of Things (IoT) and industrial automation applications [48]. Artificial neural networks (ANNs) modeling has increased acceptance as an exciting approach for predicting and real-time monitoring of stored food quality parameters [49]. These models are a set of computing algorithms that can solve difficult problems or establish complex relationships between variables by simply simulating human brain techniques. The ANNs are important because of their unique information processing qualities, including nonlinearity, noise and fault tolerance, and learning and generalization [50].

The application of ANNs in food technology has an inclusive scope. It can convert electrophoretic focusing patterns and chromatographic and spectrum data into meaningful information for predicting various food products’ functioning, physical, chemical, sensory, and rheological properties [51]. The ANNs, unlike other modeling techniques such as multilinear regression (MLR), can predict various parameters using multiple variables. Furthermore, these models differ from traditional modeling methods in that they can learn about the operation being represented without knowing the input variables or output parameters. Therefore, ANN applications are considered useful food quality and safety tools, such as modeling microbial growth; interpreting spectroscopic data; and predicting the food safety, physiochemical, sensory, and functional properties of food products during processing, storing, and distribution. In addition, these models are innovative techniques that offer a lot more potential for complicated modeling tasks in simulation and process control for food safety and quality management [52].

This study aimed to develop and evaluate an Artificial Neural Networks (ANNs) model to predict the most important physicochemical properties of date palm fruits, i.e., the pH, total soluble solids, moisture content, and water activity during cold storage based on their electrical properties.

## 2. Materials and Methods

### 2.1. Sampling Material

Ten cultivars of high-quality date palm fruits at Tamar stages (brown color, full ripening) were selected, i.e., Ruziez, Khodry, Khalas, Sagai, Sukkari, Sullag, Medjool, Sheshi, Ajwa, and Rushodiya. The date palm fruits were obtained from the orchard of Research and Training Station, King Faisal University, Al-Ahsa, Saudi Arabia (Latitude: 25°16′19.0344″ N, Longitude: 49°42′25.8228″ E) and from the Dates Packing Plant Al-Ahsa, Date Palm Research Center Al-Ahsa, Ministry of Environment, Water, and Agriculture, Kingdom of Saudi Arabia (Latitude: 25°27′54.75″ N, Longitude: 49°33′49.51″ E).

The obtained fruits were cleaned, air-dried, and then placed into aerated plastic containers (65 cm × 14 cm × 21 cm) and left at room temperature (25 °C) for 24 h. Then, they were transferred for storage in a cold storage room (48.8 m^3^ total capacity) with a set-point temperature of 5 °C [5] at the Date Palm Research Center of Excellence, King Faisal University, Saudi Arabia.

### 2.2. Physicochemical Properties Determination

The physicochemical properties, i.e., the pH, total soluble solids (TSS, Brix), moisture content (MC, %), and water activity (a_w_) of the different cultivars of date palm fruit, were measured at 0, 2, 4, and 6 months of cold storage. These quality parameters of date palm cultivars were determined in laboratories at the Date Palm Research Center Al-Ahsa, Ministry of Environment, Water, and Agriculture, Saudi Arabia. The measurements were carried out on 20 samples from each date fruit cultivar (10 cultivars) at four cold storage times (0, 2, 4, and 6 months). The total measured samples were 800 from all selected cultivars before and after the cold storage of date fruits. Therefore, 200 samples were randomly selected for measuring at each target storage time. The physicochemical properties, i.e., the pH, total soluble solids (TSS), water activity (a_w_), and moisture content (MC) of the different cultivars of date palm fruit, were measured according to AOAC analysis methods [53].

The pH of the date palm fruit was determined using a pH meter (Model HI-99121, Hanna Instruments, Leighton Buzzard, Bedfordshire, UK). Next, the TSS of the date palm fruits was determined using a digital refractometer (Model 614 RFM 840, Richmond Scientific Ltd. Unit 9, Lancashire, UK). The TSS results were expressed as Brix at 25 °C. Next, the a_w_ of date fruits was determined using a portable water activity device (Model Aqualab Series 3, Decagon Devices, Inc., Pullman, WA, USA). Finally, the MC of the fruit was determined using a portable electronic moisture balance (Model MOC-120H, Shimadzu Corporation, Kyoto, Japan.

### 2.3. Electrical Properties Determination

**Figure 1:**
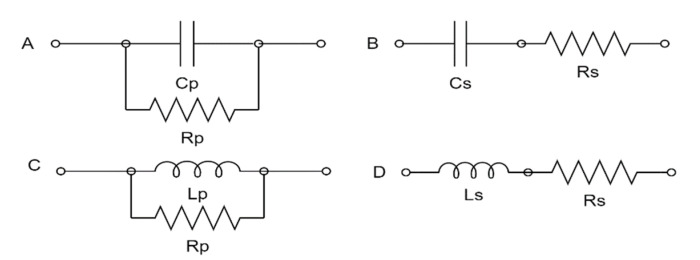
The equivalent circuits. (**A**) Resistor with a capacitor in a parallel circuit, (**B**) resistor with a capacitor in a series circuit, (**C**) resistor with a coil in a parallel circuit, and (**D**) resistor with a coil in a series circuit.

**Figure 2:**
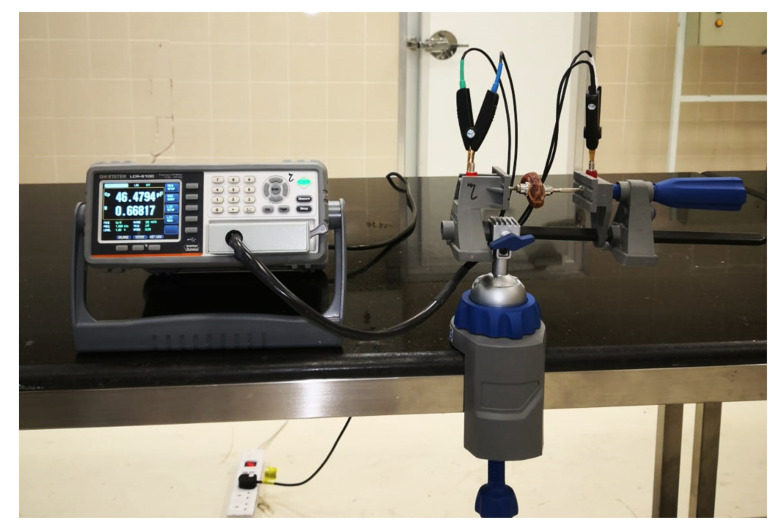
Photographic view of the measurement system to determine the electrical properties of the tested date fruits.

The electrical properties of the same fruits were nondestructively measured in intact conditions at room temperature (25 ± 0.5 °C) before they were destructed for measuring the physicochemical properties. These electrical properties of date palm cultivars were determined in the post-harvest laboratories of the Date Palm Research Center of Excellence, King Faisal University, Al-Ahsa, Saudi Arabia. A high-precision LCR (inductance, capacitance, and resistance) meter (Model Instek LCR-6100, 10 Hz–100 kHz, Good Will Instrument Co., Ltd., Tucheng Dist., New Taipei, Taiwan) was used to measure the electrical properties of the same fruits selected to measure the physicochemical measurements (800 samples) at 10, 100, 1000, 10,000, and 100,000 Hz. The measured electrical properties were the capacitance value at the series equivalent circuit model (Cs, nF), the equivalent series resistance (Rs, kΩ), the dissipation factor (D), the capacitance value at the parallel equivalent circuit model (Cp, nF), the equivalent parallel resistance (Rp, kΩ), the inductance value in the parallel equivalent circuit model (Lp, H), the inductance value in the series equivalent circuit model(Ls, H), the resistance (R, kΩ), the reactance (X, kΩ), the direct current resistance (DCR, kΩ), the absolute value of the impedance (Z, kΩ), the phase radian (θ, rad), the phase angle (θ°, degree), and the quality factor (Q). The LCR meter calculates θ and Z by measuring the electrical current flowing to the fruit being measured and the voltage across the applied electrodes. It then calculated the measurement parameters, i.e., Cs, Rs, D, Cp, Rp, Lp, Ls, R, X, DCR, and Q values. The equations employed to calculate these measurement parameters differ depending on whether the LCR meter operates in a parallel equivalent circuit mode or series equivalent circuit mode. In the case of series and parallel circuits, Figure 1 illustrates the measured electrical properties and equivalent circuits. Figure 2 shows the components of the date fruits electrical parameters measuring system. The system consists of a high precision LCR Meter, a plastic clamp with two copper electrodes, Instek LCR-06, a test lead with an alligator clip connected with the LCR meter, and a laptop. As the dielectric material, dates were placed between two conductive plate electrodes made of copper. The parameter values of electricity are monitored within a frequency range of 10 Hz to 100 kHz. The signal’s input voltage was 1 volt (RMS). Each sample was measured three times and then the average was calculated. The measurements of the electrical parameters were grouped based on the physicochemical properties of date fruits to determine the correlation and regression model.

### 2.4. Structure of Artificial Neural Networks

**Figure 3:**
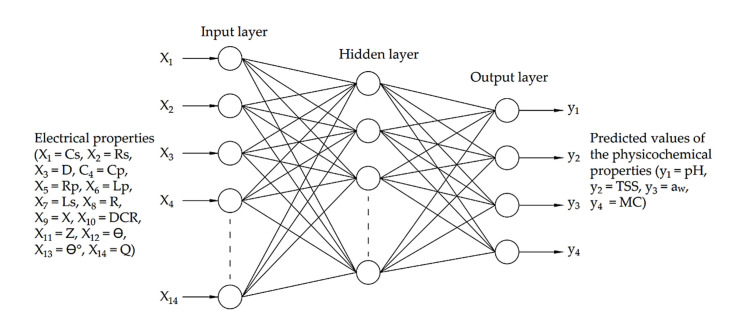
Artificial neural network architecture.

In this study, ANNs have been used as an alternative approach to predict date fruit quality attributes, i.e., the pH, TSS, a_w_, and MC, of date fruits during cold storage, which are based on 14 electrical properties (i.e., Cs, Rs, D, Cp, Rp, Lp, Ls, R, X, DCR, Z, θ, θ°, and Q). The block diagram of the applied ANNs prediction model is shown in Figure 3. The input layer acquires data obtained from the electrical properties measurement system. The hidden layer performs the data processing, and the output layer creates the continuous predicted values of the target physicochemical property. The values acquired from the input layer joined to a hidden node are multiplied by their weights, a set of predetermined values, and the outcomes, which are added to create a new value. Finally, the created value is passed as an argument to a mathematical function and an activation function to predict the target property values.

**Figure 4:**
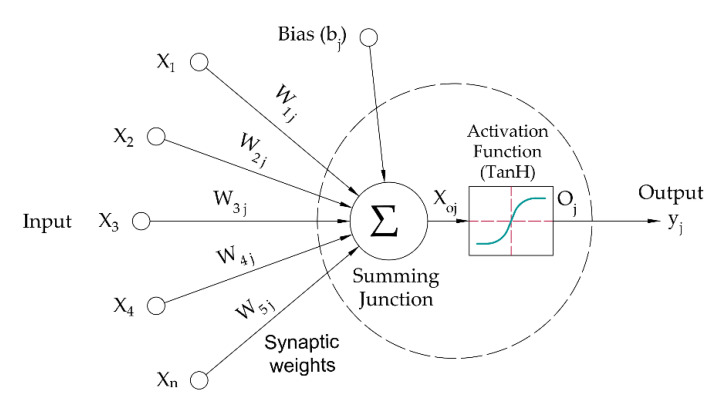
Artificial neural network active node.

The sum of the weighted inputs entering a neural network node j and the output activation function converts a neuron’s weighted value to its hyperbolic tangent (TanH) output activation function, as shown in Figure 4. The summation and the activation functions can be expressed by the following equations:

(1) $$X_{\mathrm{oj}}=\overset{n}{\underset{i=1}{\sum }}X_{i}W_{\mathrm{ij}}$$

(2) $$O_{j}=\frac{2}{1+e^{-2X_{oj}}}-1$$

where X_oj_ is the outcome of the sum, X is the input value with i as the number of the inputs, W is the weight of the input weight, and $O_{j}$ is the neuron output.

The error function depends on the values of the weights, which need to be adjusted to minimize this error. For example, for a training set (X1, t1), (X2, t2), …, (Xn, tn) that consisted of k ordered pairs of n-inputs and m-outputs (the input and output patterns), the error for the neuron output and the error function of the minimizing network error can be expressed by the following equations:

(3) $$E_{j}=\frac{1}{2}{\left(O_{j}-t_{j}\right)}^{2}$$

(4) $$E_{j}=\frac{1}{2}\overset{k}{\underset{j=1}{\sum }}{\left(O_{j}-t_{j}\right)}^{2}$$

where E_j_ is the error, $O_{j}$ is the output created by the input pattern from the training set, and $t_{j}$ is the target value.

Bias, denoted by bj in Figure 4, has either an increasing effect or lowering of the net input of the activation function. Increasing the learning rate of the ANNs model accelerates the convergence around the optimal solution—then the convergence becomes impossible. Once a set of acceptable weights has been determined, the ANNs model can use another dataset with unknown output values to automatically predict the related outputs.

For conducting this study, the multilayer perceptron module of IBM SPSS Statistics 26 (IBM Corporation, Armonk, NY, USA) was used to develop ANNs models and evaluate their accuracy. The multilayer perceptron module neural networks are trained with a backpropagation learning algorithm that uses gradient descent to update the weights toward minimizing the error function. The data were randomly set to 60% for training, 20% for testing, and 20% for holdout subsets. The training dataset was used to determine the weights and construct the model; the testing data was used to determine the errors and stop overtraining in the training mode. Finally, the holdout data was used to validate the prediction ANNs model.

### 2.5. Multilinear Regression

In this study, the multilinear regression (MLR) was used to develop a model for predicting date fruit quality attributes, i.e., the pH, total soluble solids (TSS), water activity (a_w_), and moisture content (MC), of the fruits during cold storage, which is based on 14 electrical properties (i.e., Cs, Rs, D, Cp, Rp, Lp, Ls, R, X, DCR, Z, θ, θ°, and Q). The MLR function is a linear equation that can be expressed in the following formula:

(5) $$y_{i}=B_{0}+\overset{n}{\underset{i=1}{\sum }}B_{i}x_{i}$$

(6) $$y=B_{0}+B_{1}x_{1}+B_{2}x_{2}+\ldots +B_{n}x_{n}$$

where y_i_ is the dependent variable, i is the variable’s number (n), $x_{i}$ is the independent variable, B_0_ is the constant of the y-intercept, and B_i_ is the constant of the slope coefficients for each explanatory variable. The constant of the regression equations for the pH, TSS, a_w_, and MC were determined using IBM SPSS Statistics 26 software.

### 2.6. Statistical Analysis and ANNs Evaluation

After measuring the physicochemical and electrical properties of different cultivars of date fruits, the data were analyzed using IBM computer software SPSS Statistics, version 26. A Tukey test was used at a 5% probability level to separate mean differences. The values of the coefficient of determination (R^2^) and the root-mean-square error (RMSE) were used to evaluate the performance of the prediction MLR and ANNs models at the various testing frequencies. The criteria of R^2^ and RMSE can be expressed as follows:

(7) $$R^{2}=1-\frac{\sum {\left(M_{j}-T_{j}\right)}^{2}}{\sum M_{j}^{2}-\frac{{\left(\sum M_{j}\right)}^{2}}{n}}$$

(8) $$\mathrm{RMSE}=\sqrt{\frac{\sum_{j}^{n}{\left(M_{j}-T_{j}\right)}^{2}}{n}}$$

where M_j_ and T_j_ are the measured and the predicted values, respectively, of data j, and n is the number of the measurement.

## 3. Results and Discussion

### 3.1. Physicochemical Data

Fruit pH showed a statistically significant (*p* ≤ 0.05) difference among date palm cultivars, and cv. Khalas had the highest fruit pH, followed by Sukkari, Sheshi, Khodry, and Sullag (Table 1). Similarly, the TSS was maximum in cv. Khalas and minimum in cv. Salag. The lowest moisture content was recorded in cvs. Rushodiya and Sukari, which were statistically at par, whereas it was highest in cv. Sagai. The highest range of water activity was determined in cvs. Rushodiya and Sagai, followed by Khadri, which significantly decreased in cvs. Ruziez, Medjool, and Ajwa.

Previous studies have demonstrated that various date cultivars have considerable variations in fruit physicochemical attributes [23,54,55,56]. The pH variations can affect the fruits’ flavor, aroma, texture, and shelf life due to organic acids, which change from cultivar to cultivar. Since pH levels greater than 4.6 indicate low-acidic values, the current investigation found that all date palm cultivars at the Tamar stage exhibited low-acidic qualities. It has already been reported in a few other studies that the fruit pH decreases with maturity stages, and it is lowest at the final stage of ripening [57,58,59]. In general, the increase in the TSS value is related to the decrease in the moisture content of the fruit [60,61,62].

In this study, it was observed that each cultivar of the ten tested cultivars responded individually. For example, the TSS value in cv. Khalas was 71% higher than the moisture content, which means the higher TSS value of cv. Khalas might be due to the significant decrease in moisture content from the fruit surface. The moisture content of date palm fruits declines rapidly as they ripen. For example, the fruits of certain date palm cultivars that were sold as fresh at the Rutab stage have a moisture content of less than 35%. It further decreases to less than 24% when the fruit ripened at the Tamar stage and reaches 4–10% in ripened dry date cultivars [63,64]. Similarly, date fruits with a moisture content < 40% and a water activity < 0.90 are generally unsuitable for microbial growth [65].

Our results indicated 15.72–21.06% of the moisture content in the date palm cultivars studies and are categorized as semi-dry cultivars. These findings coincide with those previously reported, with a few variations due to the date palm cultivars and climatic conditions [66,67,68]. Due to its importance as an index of date quality stability and microbial spoilage, water activity must be considered in date fruit standards. A water activity of more than 0.95 generally encourages the growth of microorganisms in fruits and vegetables [69]. Nadeem et al. [70] reported that many local and commercial date palm cultivars had a 0.32 to 0.48 water activity. Aleid et al. [8] reported a non-significant difference in water activity in date palm cultivars, i.e., Sukkary, Khalas, Sugai, and Anbara, ranging from 0.417 to 0.623. They were studied on date palm cvs. Aseel, Dhakki, Karbalain, Muzawati, and Rabai, Rashid et al. [71] determined the lowest water activity (0.680) in cv. Aseel and highest (0.795) in cv. Rabai. They stated that the product deterioration risk is minimal in cv. Aseel because of low water activity. In the present study, although the water activity in all date palm cultivars is lower than 0.95, cys. Ruziez, Ajwa, Medjool, Sullag, Sheshi, and Sukkari cultivars have minimal risk of microbial spoilage.

Fruit pH was significantly decreased when date palm fruits were stored for different durations (Table 2). It was higher in unstored fruits (6.07), which decreased to 5.69 after 6 months of storage. Fruit TSS, moisture content, and water activity showed the opposite trend to pH, as these attributes increased with the increase in storage duration. Unstored fruits had a lower TSS (42.32 Brix), moisture content (17.05%), and water activity (0.49) compared to fruits stored for 6 months, which had a higher TSS (64.11 Brix), moisture content (19.49%), and water activity (0.64).

Hazbavi et al. [72] mentioned that, after storing dates (cv. Stamaran) for six months of storage, the pH was reduced by 5.4%. A 10.97% decrease in the pH of the date palm cv. Tamar fruits of date palm (cv. Khalas) stored at −18 °C for 6 months had the lowest pH compared to Khalal and Rutab fruits [7]. Aleid et al. [8] reported that fruit pH decreased while TSS and moisture content increased when the date fruits (cv. Khalas) were stored at 5 °C for 12 months.

The findings of the present study showed a 6.26% reduction in the fruit pH after 6 months of storage. Microorganisms’ fermentative activity results in the production of organic acids and a decrease in pH [73]. According to our study, the TSS of date palm fruits stored for 6 months increased by 51.48%. Similarly, an increase in TSS was observed in date palm cv. Barhi, which was stored for 70 days at 0 °C and 90–95% RH [60]. The TSS increased in Tamar fruits of date palms (cv. Mazafati) stored for 180 days at 4 °C [19]. The enzymatic conversion of large polysaccharides into small sugars would be the main reason for the increase in TSS [72]. Radi et al. [74] suggested that the increase in TSS of date fruits during storage could be related to microbial and enzymatic activities degrading high molecular weight compounds to low molecular weight ones.

Our study also showed that the moisture content and water activity of date palm fruits were increased by about 14.31% and 30.61%, respectively, after 6 months of storage. Mohammed et al. [14] recorded a 19.05% moisture content and 0.76 water activity in date fruits (cv. Khalas) stored at 5 °C and 80% RH. Another study indicated that the moisture content was not significantly increased in cvs. Majhoul and Boufeggous, whereas TSS was increased in cv. Majhoul after 5 months of storage at 2–4 °C and 66–68.5% RH [75]. The evaporation of fruit water caused by the relatively high temperature and moderate RH could explain the decrease in both moisture and water activity.

### 3.2. Electrical Properties Data

The electrical properties of date palm fruits were determined at different frequencies and were significant at *p* ≤ 0.05 (Table 3). The measured electrical properties, such as the capacitance value (series equivalent circuit model), equivalent series resistance, dissipation factor, capacitance value (parallel equivalent circuit model), equivalent parallel resistance, resistance, and the absolute value of impedance, were very large at a low frequency (10 Hz). However, the reactance, phase radian, and phase angle electrical properties were higher at 100 Hz. As a result, the inductance value (series equivalent circuit model) was a maximum at 1000 Hz frequency, while the inductance value (parallel equivalent circuit model) was measured as higher than 100,000 Hz. The statistical difference was non-significant regarding the direct current resistance parameter; however, its value was linearly increased with the increase in frequency.

Due to the high electrical capacity of cell membranes, electrical current only flowed via extracellular fluid, which has a relatively high resistance in the low-frequency area. However, the impedance drops significantly in the high-frequency range because the current can travel through intracellular fluid, which has a low resistance. It can be seen that, as the frequency increased, the impedance decreased dramatically. Dispersion is a phenomenon in which the decrease in impedance is proportional to the increase in frequency [46]. As the frequency increased, the capacitance of the cell membrane dropped. The amount of water in biological cells is critical for their structural and functional integrity [42]. As a result, it is supposed that when fruit moisture is reduced, structural changes in the cell membrane cause a decrease in cell membrane capacitance [43]. The ion efflux across the fruit membrane produced by osmotic changes in the extracellular fluid modified the electrical properties of the cell membrane. Thus, alterations in cell membrane capacitance could have been created by ion movement caused by plasmolysis when moisture is declined [34,43]. Heat injury to plant cells causes a reduction in the capacitance of the cell membrane [40].

At low temperatures, electrode polarization was shown to account for a higher fraction of overall impedance in potatoes, but extracellular resistance and capacitances continued to decline. It could be the electrolyte leakage to the extracellular space, presumably due to membrane injury [76]. Soltani et al. [77] applied a capacitance sensing system to predict banana quality during ripening and found a good relationship between SSC and firmness at a 1 MHz frequency. Similarly, the highest inductive value was observed at 34 MHz frequency to determine the ripening time of oil palm fruit bunch [78]. The electrical impedance values of various apple cultivars rapidly decreased, which is assumed to correlate to dispersion induced by cell membrane capacitance [39,41]. Electrical current flows through the extracellular fluid at low frequencies, thereby avoiding cell membranes. At high frequencies, however, the cell membranes act as a conductor. The current flows through the intracellular fluid, which contains more electrolytes; thus, the impedance at low frequencies is higher [39,79].

Table 4 shows that the average values of important electrical properties of date palm fruits were significantly (*p* ≤ 0.05) varied at different storage durations (0, 2, 4, and 6 months). The fruits before storage showed higher values regarding series resistance, dissipation factor, parallel resistance, resistance, direct current resistance, the absolute value of impedance, phase radian, and phase angle. However, there was a non-significant difference in the phase radian and phase angle parameters between before storage and 2 months of storage fruits. The maximum quality factor value was observed in fruits stored for 4 months, followed by 6 months of storage fruits and before storage fruits. The capacitance value (series equivalent circuit model), capacitance value (parallel equivalent circuit model), inductance value (parallel equivalent circuit model), inductance value (series equivalent circuit model), and reactance were maximal after 6 months of cold storage fruits. However, there was a non-significant difference in the capacitance value (series equivalent circuit model) and capacitance value (parallel equivalent circuit model) parameters between 4 and 6 months of stored fruits.

Different researchers reported that fruit electrical conduction increases with temperature, field strength, storage duration, sugar concentration, and fruit firmness [38,80,81,82,83,84]. Watanabe et al. [39] stated that the initial resistance and reactance values of apple fruit varied by cultivar and declined as storage duration increased. The non-uniformity of electrical characteristics was attributed to non-uniform conditions, such as differences in cell size or shape between cultivars. They also stated that the LTO, which relates to the resistance of the extracellular parts of the fruit, declined after four weeks of storage and then increased. However, it decreased after 16 weeks of storage in some apple cultivars. Our study indicated that resistance decreased with the increase in storage duration. Extracellular resistance has been reported to decrease in vegetables due to the degradation of cell membrane integrity during storage [44,85]. It is assumed that the electrical resistance increase in apples after storage was caused by water transpiration during storage [86]. The water activity and moisture content of apples significantly impacted dielectric characteristics during storage. Therefore, the change in electrical parameters can be used to indirectly reflect changes in internal quality [35]. Jiangjie et al. [87] found that a frequency of 39.8 kHz could be used for nondestructive post-harvest quality detection for new red star apples, and that the absolute value of impedance, equivalent parallel resistance, and capacitance could be used as sensitive electrical parameters to indicate quality parameter changes during the ripening and senescence. According to Sastry [88], electrical conduction increases with storage duration and the difference was negligible when the temperature is changed. In the present study, electrical parameters such as capacitance, dissipation factor, inductance, and reactance increased with the increased storage time.

### 3.3. Correlation between Physicochemical and Electrical Properties

Table 5 shows the correlation between the fruit physicochemical traits, such as pH, TSS, moisture content, and water activity. The electric parameters at various frequencies vary significantly, either positively or negatively. At a low frequency (10 Hz), there was a significant positive correlation between pH, inductance value (parallel), and inductance value (series); and TSS, moisture content, and water activity with capacitance value (parallel) and quality factor. Similarly, at 100 Hz frequency, pH had a strong positive correlation with dissipation, phase radian, phase angle, and quality factor. The electrical parameters, inductance value (parallel) and inductance value (series), had a better positive correlation with TSS and moisture content.

In contrast, water activity positively correlated with inductance value (parallel) at 1000 Hz frequency. The electric parameter reactance was positively correlated with pH and moisture content at a 10,000 Hz frequency. At a high frequency (100,000 Hz), fruit pH was positively correlated with equivalent series resistance, capacitance value (parallel), equivalent parallel resistance, resistance, and the absolute value of impedance; fruit TSS with capacitance value (series) and reactance; moisture content with capacitance value (series), phase radian, and phase angle; and water activity with capacitance value (series), dissipation factor, inductance value (series), reactance, phase radian, and phase angle.

The electrical resistance showed a significant decline as the citrus fruit matured and was closely correlated to the changes in pH [89]. Our results showed a negative correlation of resistance when correlated with pH, TSS, moisture content, and water activity. However, it positively correlated with pH when the frequency was 10,000 and 100,000 Hz. A reduced resistance is also linked to a reduction in hardness and TSS rise [90]. Soltani et al. [77] found a correlation between the soluble solid contents, firmness of the fruit, and the capacitance sensing system parameters. There was a strong correlation between the quality parameters and the relative permittivity of the capacitive property. Citrus tissues found a linear relationship between the matrix moisture content and the dielectric constant γ-relaxation was found in citrus tissues. They indicated that the dielectric constant under γ-relaxation is an important tool for predicting the moisture content of citrus fruit [91]. The highest correlation with various frequencies predicted the quality parameters of the damaged apples [92]. The cell wall, membranes, and composition of the cell contents may all undergo significant modifications during storage. All these alterations would have an impact on tissue capacitance. A change in reactance can be used to indicate changes in capacitance. The impedance values will change when the resistance and reactance parameters change. As a result, its relationship with physicochemical properties will be comparable. During the storage of date fruits, the value of resistance and reactance decreased, lowering the value of its impedance [93].

### 3.4. ANNs and MLR Models

The ANNs technique in SPSS was adopted to determine the optimal prediction model at different testing frequencies. The number of hidden layers was one for all ANNs at the various testing frequencies. The input layer in the ANNs at 10 and 100 Hz contains 13 neurons for the independent variables (Cs, Rs, D, Cp, Rp, Ls, R, X, DCR, Z, ϴ, ϴ°, and Q), and the optimal hidden layers contain 12 neurons at 10 Hz and 14 neurons at 100 Hz. The input layer in the ANNs at 1000, 10,000, and 100,000 Hz contains 14 neurons for the independent variables (Cs, Rs, D, Cp, Rp, Ls, Lp, R, X, DCR, Z, ϴ, ϴ°, and Q), and the optimal hidden layers contain 15 neurons. The output layer contains four neurons for the dependent variables (pH, TSS, a_w_, and MC). The rescaling method for covariates was standardized. The activation function applied for the hidden layers was a hyperbolic tangent. The activation function applied for the output layers was Identity for all ANNs models at the different testing frequencies. About 60% of the measured data were used as a training dataset, 20% for model testing, and 20% for evaluation. The sum of squares was used as an error function because of the Identity function.

**Figure 5:**
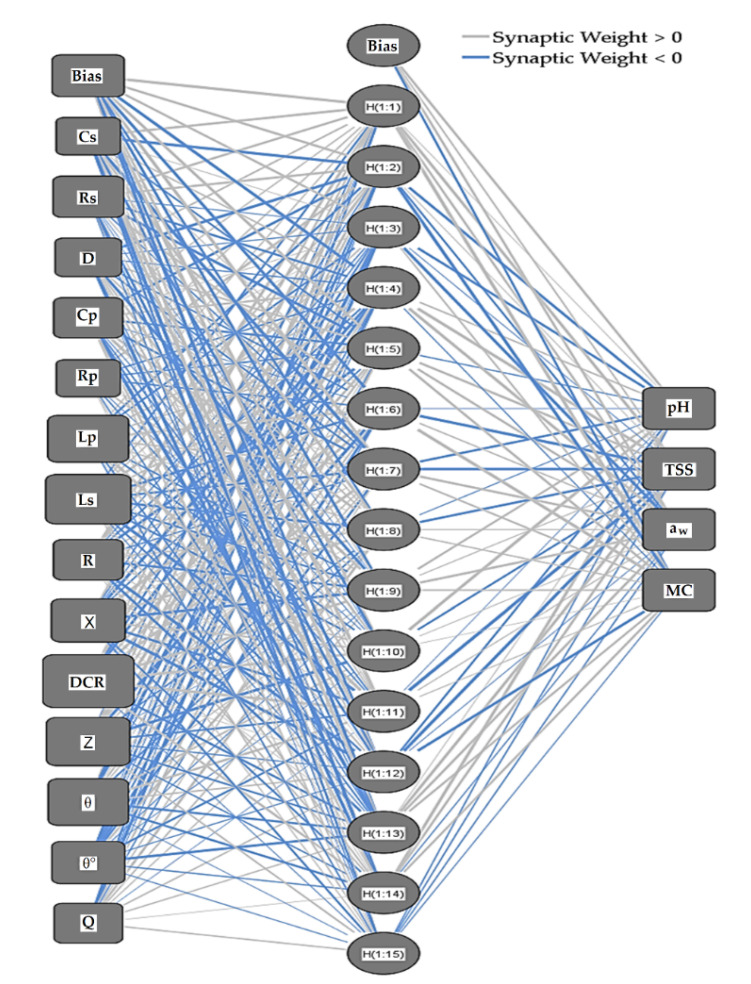
Network diagram of the ANNs prediction model to predict the pH, TSS, a_w_, and MC of the date fruits during cold storage based on their electrical properties (Cs, Rs, D, Cp, Rp, Ls, Lp, R, X, DCR, Z, ϴ, ϴ°, and Q) at 10,000 Hz. The hidden layer activation function is Hyperbolic tangent, and the output layer function is Identity.

Figure 5 shows the optimal ANNs diagram applied to predict the pH, TSS, a_w_, and MC of the date fruits during cold storage based on their electrical properties at 10,000 Hz. The diagram shows the 14 input nodes (Cs, Rs, D, Cp, Rp, Ls, Lp, R, X, DCR, Z, ϴ, ϴ°, and Q), the 15 hidden nodes, and the 4 output nodes representing the predicted values of the target physicochemical properties (pH, TSS, a_w_, and MC). The trained ANNs quickly determine the target physicochemical properties when fed the system’s electrical properties data.

**Figure 6:**
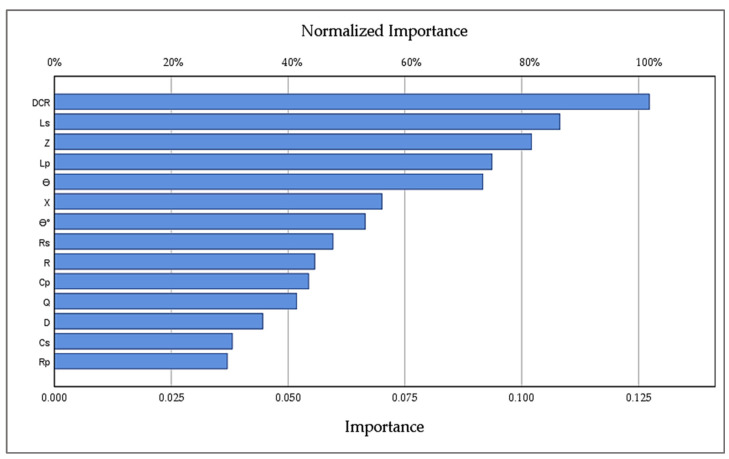
Importance of the independent variable electrical properties (Cs, Rs, D, Cp, Rp, Ls, Lp, R, X, DCR, Z, ϴ, ϴ°, and Q) at 10,000 Hz.

Figure 6 shows the importance of the independent variables (Cs, Rs, D, Cp, Rp, Ls, Lp, R, X, DCR, Z, ϴ, ϴ°, and Q) at 10,000 Hz in the ANNs model in terms of the relative and normalized importance. This figure displays the impact of the change of each independent variable on the ANNs prediction model. The variables related to DCR, Ls, Z, Lp, ϴ, X, and ϴ° have the most critical effect on how the network predicts the values of the dependent variables, i.e., pH, TSS, a_w_, and MC at 10,000 Hz.

Table 6 displays the comparison between error results of the ANNs models, i.e., the sum of squares error, average overall relative error, and relative error in the training, testing, and holdout phases at various testing frequencies. In addition, relative errors were displayed depending on the dependent variables of the pH, TSS, a_w_, and MC measurement levels. From Table 1, it is noticed that the values of the electrical parameters at 10,000 Hz gave better results regarding the model errors in the phases of training, testing, and holdout datasets. Based on these results, adopting electrical measurements of dates at 10,000 Hz can successfully predict the pH, total soluble solids (TSS), water activity (a_w_), and moisture content of the date fruits during cold storage.

The R^2^ and RMSE related to the MLR models for predicting the pH, TSS, a_w_, and MC parameters under the testing frequencies are presented in Table 7. The F-test showed that several independent variables in the MLR for pH property are significant (*p* ≤ 0.05) at 10,000 Hz.

The MLR prediction models for pH values based on the electrical parameters at 100 Hz (R^2^ = 0.813, RMSE = 0.289), 1000 Hz (R^2^ = 0.842, RMSE = 0.176), 10,000 Hz (R^2^ = 0.843, RMSE = 0.175) are characterized by a significative determination coefficient, which can be used as a significative predictive model at one of these frequencies. The other MLR models were characterized by low R^2^ and high RMSE values. Therefore, the MLR models are considered unsuitable for accurately evaluating the TSS, a_w_, and MC.

The prediction models using the MLR technique based on the electrical properties measured by a 10,000 Hz frequency are the best for all target properties of pH, TSS, a_w_, and MC.

The developed MLR prediction models for pH, TSS, a_w_, and MC at 10,000 Hz that acquired the best results are given below:

pH = 6.146 − 0.058 × Cs + 0.003 × Rs + 0.105 × D + 0.155 × Cp − 0.035 × Lp + 0.008 × Ls − 0.013 × X + 0.001 × DCR − 0.01 × Z − 0.126 × ϴ + 0.064 × ϴ° + 1.949 × Q

TSS = 57.303 + 0.788 × Cs − 0.062 × Rs − 2.151 × D + 1.784 × Cp − 1.755 × Lp + 0.413 × Ls − 0.544 × X − 0.104 × DCR − 0.09 × Z − 231.5 × ϴ + 3.51 × ϴ° + 2.749 × Q

a_w_ = 0.583 + 0.006 × Cs + 0.0001 × Rs − 0.011 × D + 0.011 × Cp − 0.006 × Lp + 0.001 × Ls − 0.001 × X + 0.0001 × DCR − 0.001 × Z + 1.283 × ϴ − 0.027 × ϴ° − 0.114 × Q

MC = 23.5 + 0.052 × Cs − 0.003 × Rs − 0.915 × D + 0.011 × Cp + 0.134 × Lp − 0.031 × Ls + 0.065 × X + 0.014 × DCR + 0.009 × Z − 36.349 × ϴ + 0.869 × ϴ° + 0.114 × Q

The performance of the ANNs and MLR prediction models at various frequencies based on R^2^ and RMSE values in the evaluation set is shown in Table 7. The high R^2^ and low values of RMSE indicated that the ANNs models present promising possibilities to predict the target physicochemical properties of date fruits based on their electrical properties. Based on R^2^ and RMSE in Table 7, it is shown that the ANNs modeling techniques were more efficient compared with the MLR models for predicting the pH, TSS, a_w_, and MC values at all testing frequencies. The results showed that the R^2^ for ANNs models at frequencies of 1000, 10,000, and 10,0000 Hz was more acceptable than the ANNs models at 10 and 100 Hz. The high values of R^2^ were obtained for pH (R^2^ = 0.938), TSS (R^2^ = 0.954), a_w_ (R^2^ = 0.876), and MC (R^2^ = 0.855) in the evaluation set of ANNs models based on the measured electrical properties of the stored date fruits at 10,000 Hz. Furthermore, the low values of RMSE were obtained also at 10,000 Hz for the pH (RMSE = 0.121), TSS (RMSE = 2.946), a_w_ (RMSE = 0.020), and MC (RMSE = 0.803) in the evaluation set of ANNs models.

Generally, based on these results, the MLR models had lower performance and weaker predictive ability than ANNs for predicting pH, TSS, a_w_, and MC at various frequencies (Table 7). Based on these results, the MLR models are unsuitable for predicting the target properties and show a relative disadvantage because they only describe the linear relationship between variables.

**Figure 7:**
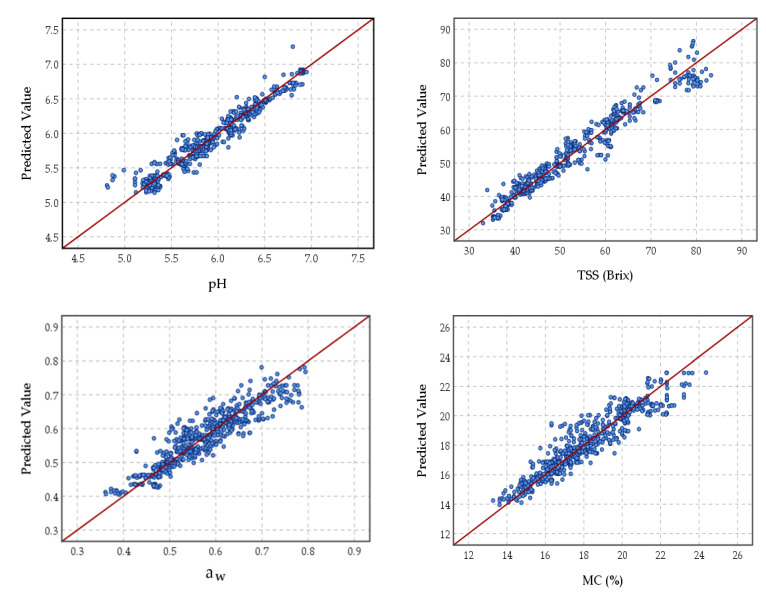
Scatter plots of measured values of pH, total soluble solids (TSS), water activity (a_w_), and moisture content (MC) versus the predicted values by the neural networks model in the evaluation phase based on the measured electrical properties of the stored date fruits at 10,000 Hz.

Figure 7 presents the scatter plots of the measured values of pH, TSS, a_w_, and MC of the stored date fruits versus the predicted values by the neural networks model in the evaluation phase based on the measured electrical properties of the stored date fruits at 10,000 Hz. The network structure of the model was one hidden layer and 14 neurons, which exhibited the highest level of accuracy. The prediction error in the training and testing phases at 10,000 Hz was lower than in the same phases at other frequencies. The results displayed that the ANNs model at 10,000 Hz was more accurate than the various frequencies in the evaluation phase. The regression line between the predicted and the observed values of the target properties, i.e., pH (y = 0.38 + 0.94 x), TSS (y = 1.87 + 0.96 x), a_w_ (y = 0.09 + 0.84 x), and MC (y = 2.36 + 0.87) at validation sets, nearly overlapped the 1:1 line (y = x + 0).

So far, to our knowledge, there is no study that has employed the feed-forward ANNs with a backpropagation training algorithm for the prediction of the physicochemical properties of date fruits based on their electrical properties. Nayak et al. [48] mentioned that the dates are the type of fruit that have been used very rarely to process with ANNs. However, Fadel suggested a novel method for classifying dates using Probabilistic Neural Networks (PNN) based on the color of five cultivars of date fruits. The authors observed good classification accuracy in the experimental process [94]. Hsu et al. [95] mentioned that the ANNs learned to find the solutions for the problem by developing a memory capable of associating many input patterns with a resulting set of effects or outputs. The problem with these ANNs models is the dependency on data for their training. The training phase of the models is the process of updating the internal representation of the model in response to external variables to achieve a specific task. In addition, it modifies the network architecture, which involves modifying the weights of the links, changing connection links by removing or creating new links, and changing the individual neurons firing rules [96]. Sablani and Rahman proposed [49] an ANNs model to predict the thermal conductivity of food, i.e., apple, corn starch, pear, raisin, potato, starch, ovalbumin, sucrose, carrot, and rice, as a function of moisture content, apparent porosity, and temperature. The optimal proposed ANNs model consisted of two hidden layers with four neurons in each layer. This model predicted the thermal conductivity with low mean absolute and relative errors. Singh [97] has proposed a methodology for the sweet potatoes during drying using an ANNs model to get online predictions of moisture kinetics in the potatoes. The results achieved in their work showed that the predicting ANNs model with two hidden neurons and a feed-forward network could help envisage and model the moisture relocate in the product.

Our results indicated that the MLR models were less accurate than the ANNs models for the prediction of fruit pH and had low performance and weak predictive ability for the TSS, a_w_, and MC at all testing frequencies. If the constants or parameters of a mathematical equation that relates the input variables to the output variable are defined for a given mathematical equation that relates the input variables to the output variable, the difference between the predicted output and the observed output of the equation for the set of input data is a minimum for statistical regression, such as the MLR. As a result, ANNs can be used to study ambiguous and unclear datasets and their interactions, but statistical regression analysis, i.e., MLR, will fail in such cases. Furthermore, ANNs can be used to analyze more data at the same time with more complicated and complex interactions. Even if the data is incomplete and noisy, ANNs can outperform MLR in prediction, modeling, and optimization [52]. Therefore, MLR models were unsuitable for our research, whereas the ANNs prediction model accurately predicted the quality attributes we were looking for. The results of ANNs are simple and do not require any modifications. ANNs are several types of intelligent modeling techniques that can solve a problem by analyzing scarce, unstructured, and incomplete numerical data about non-stationary and nonlinear systems [98]. The ANNs developed a solution by training on their measurements using the nonlinear correlation between various variables.

This study indicated that the ANNs model was found to be a powerful tool for efficiently predicting the pH, TSS, water activity, and moisture content of date fruits based on their electrical properties.

## 4. Conclusions

This study indicated that the ANNs model is a powerful tool to efficiently predict the date fruits’ quality based on their electrical properties during cold storage. The model established a new solution based on measured data using the nonlinear correlation between numerous variables. The optimal developed ANNs model had a 14-neuron input layer (electrical properties), a 15-neuron hidden layer, and a 4-neuron output layer. This model enhanced the fast and easy prediction of the pH, TSS, a_w_, and MC of stored date fruits during cold storage with high R^2^ and low RMSE. The MLR models, on the other hand, were less accurate than ANNs models in predicting fruit pH and had poor performance and prediction abilities for the TSS, water activity, and moisture content across all testing frequencies. Based on these results, the MLR models are unsuitable for predicting the targeted attributes. Further research is needed to predict more chemical and mechanical properties of stored fruits based on their electrical properties, such as reducing and non-reducing sugars and texture parameters (hardness, adhesiveness, springiness, cohesiveness, gumminess, chewiness, and resilience). In addition, the quality of the stored fruits can be remotely monitored in real time using the Internet of Things (IoT) and ANNs prediction models.

## Figures and Tables

**Table 1:** Comparison of the mean values ± standard deviation of the pH, total soluble solids (TSS), water activity (a_w_), and moisture content (MC) of the ten stored date fruits cultivars.

Date Fruit Cultivars	Characteristics			
	pH	TSS (Brix)	MC (%)	a_w_
Ruziez	5.61 ± 0.32 ^G^	57.55 ± 13.73 ^B^	18.34 ± 1.31 ^D^	0.52 ± 0.12 ^F^
Khodry	6.14 ± 0.09 ^D^	56.07 ± 9.71 ^C^	20.52 ± 0.91 ^B^	0.61 ± 0.07 ^A^
Khalas	6.56 ± 0.32 ^A^	65.63 ± 6.56 ^A^	19.03 ± 2.52 ^C^	0.59 ± 0.08 ^B^
Sagai	5.77 ± 0.18 ^F^	44.42 ± 9.12 ^I^	21.06 ± 0.95 ^A^	0.62 ± 0.04 ^A^
Sukkari	6.32 ± 0.23 ^B^	51.28 ± 12.93 ^E^	15.85 ± 1.47 ^G^	0.56 ± 0.05 ^C^
Sullag	6.05 ± 0.29 ^E^	47.76 ± 14.12 ^F^	18.59 ± 1.75 ^D^	0.55 ± 0.09 ^CD^
Medjool	5.81 ± 0.24 ^F^	52.8 ± 11.71 ^D^	18.57 ± 1.32 ^D^	0.54 ± 0.06 ^E^
Sheshi	6.23 ± 0.2 ^C^	46.08 ± 3.82 ^H^	17.74 ± 1.17 ^E^	0.55 ± 0.09 ^CD^
Ajwa	5.50 ± 0.18 ^H^	46.64 ± 9.3 ^G^	16.96 ± 1.3 ^F^	0.54 ± 0.09 ^E^
Rushodiya	5.28 ± 0.13 ^I^	51.45 ± 8.98 ^E^	15.72 ± 1.18 ^G^	0.62 ± 0.08 ^A^

The means (*n* = 80) within each column with the same letters are not significantly different at *p ≤* 0.05.

**Table 2:** Comparison of the mean values ± standard deviation of the pH, total soluble solids (TSS), moisture content (MC), and water activity (a_w_) of the stored date fruits under different cold storage times.

Characteristics	Storage Time (Months)			
	0	2	4	6
pH	6.07 ± 0.45 ^A^	6.02 ± 0.42 ^B^	5.91 ± 0.4 ^C^	5.69 ± 0.41 ^D^
TSS (Brix)	42.32 ± 7.01 ^D^	46.44 ± 6.7 ^C^	54.99 ± 9.29 ^B^	64.11 ± 11.09 ^A^
MC (%)	17.05 ± 1.95 ^D^	17.68 ± 1.8 ^C^	18.74 ± 2.01 ^B^	19.49 ± 2.24 ^A^
a_w_	0.49 ± 0.06 ^D^	0.54 ± 0.05 ^C^	0.61 ± 0.07 ^B^	0.64 ± 0.07 ^A^

The means (*n =* 800) within each column with the same letters are not significantly different at *p ≤* 0.05.

**Table 3:** Comparison of the mean values ± standard deviation of the electrical parameter of the stored date fruits under various testing frequencies. Where Cs is capacitance value (series equivalent circuit model), Rs is equivalent series resistance, D is dissipation factor, Cp is capacitance value (parallel equivalent circuit model), Rp is equivalent parallel resistance, Lp is inductance value (parallel equivalent circuit model), Ls is inductance value (series equivalent circuit model), R is resistance, X is reactance, DCR is direct current resistance, Z is the absolute value of impedance, ϴ (rad) is phase radian, ϴ° is phase angle, and Q is the quality factor.

Parameters	Frequency (Hz)				
	10	100	1000	10,000	100,000
Cs (nF)	1916.73 ± 1926.05 ^A^	771.77 ± 1369.47 ^B^	40.89 ± 55.76 ^C^	2.3 ± 3.63 ^C^	0.3 ± 0.3 ^C^
Rs (kΩ)	687.33 ± 738.17 ^A^	602.25 ± 610.49 ^B^	542.33 ± 534.54 ^B^	389.32 ± 339.27 ^C^	91.1 ± 51.63 ^D^
D	28.55 ± 16.79 ^A^	20.19 ± 12.05 ^B^	8.86 ± 3.95 ^C^	3.28 ± 1.71 ^D^	1.19 ± 0.87 ^E^
Cp (nF)	136.15 ± 159.6 ^A^	31.52 ± 66.83 ^B^	2.97 ± 4.84 ^C^	1.09 ± 1.85 ^C^	0.7 ± 1.3 ^C^
Rp (kΩ)	646.73 ± 686.5 ^A^	606.4 ± 616.16 ^AB^	552.71 ± 551.47 ^B^	468.83 ± 456.8 ^C^	308.34 ± 264.8 ^D^
Lp (H)	−1422.6 ± 1641 ^C^	−1261.5 ± 2214 ^C^	−725.71 ± 592.5 ^B^	−34.98 ± 51.45 ^A^	−29.31 ± 97.31 ^A^
Ls (H)	−464.63 ± 617.51 ^C^	−86.85 ± 123.01 ^B^	−11.67 ± 14.8 ^A^	−65.5 ± 209.08 ^B^	−12.77 ± 42.96 ^A^
R (kΩ)	640.7 ± 677.13 ^A^	605.6 ± 613.19 ^AB^	547.58 ± 537.3 ^B^	391.56 ± 339.1 ^C^	94.59 ± 53.75 ^D^
X (kΩ)	−110.84 ± 330.09 ^B^	−65.09 ± 104.27 ^A^	−103.71 ± 137.53 ^B^	−163.82 ± 203.84 ^C^	−130.02 ± 103.78 ^B^
DCR (kΩ)	681.48 ± 698.49 ^A^	689.85 ± 709.44 ^A^	705.66 ± 726.77 ^A^	708.08 ± 728.74 ^A^	713.14 ± 730.99 ^A^
Z (kΩ)	642.84 ± 679.61 ^A^	614.38 ± 619.67 ^AB^	554.67 ± 547.39 ^B^	454.89 ± 452.38 ^C^	164.41 ± 111.08 ^D^
ϴ (rad)	−0.14 ± 0.12 ^B^	−0.11 ± 0.08 ^A^	−0.16 ± 0.07 ^B^	−0.41 ± 0.18 ^C^	−0.86 ± 0.26 ^D^
ϴ°	−8.17 ± 6.75 ^B^	−6.32 ± 4.48 ^A^	−9.06 ± 4.26 ^B^	−23.45 ± 10.27 ^C^	−48.74 ± 14.74 ^D^
Q	0.16 ± 0.13 ^C^	0.44 ± 1.11 ^B^	0.17 ± 0.08 ^C^	0.47 ± 0.25 ^B^	1.27 ± 0.6 ^A^

The means (*n =* 610 for Lp parameter at 10 Hz, *N* = 649 for Lp parameter at 100 Hz, *n* = 800 for Lp at 1000, 10,000, and 100,000, *n =* 800 for other electrical parameters) within each column with the same letters are not significantly different at *p* ≤ 0.05.

**Table 4:** Comparison of the mean values ± standard deviation of the electrical parameter of the stored date fruits under different cold storage times.

Parameters	Storage Time (Months)			
	0	2	4	6
Cs (nF)	287.35 ± 785.39 ^B^	418.68 ± 927.34 ^B^	682.6 ± 1459.09 ^A^	796.96 ± 1709.89 ^A^
Rs (kΩ)	725.08 ± 728.62 ^A^	515.7 ± 525.32 ^B^	400.64 ± 426.96 ^C^	208.44 ± 321.16 ^D^
D	17.46 ± 18.78 ^A^	13.17 ± 13.66 ^B^	11.49 ± 11.33 ^C^	7.53 ± 8.55 ^D^
Cp (nF)	20 ± 70.79 ^B^	27.05 ± 61.87 ^B^	42.39 ± 98.19 ^A^	48.49 ± 125.91 ^A^
Rp (kΩ)	809.45 ± 703.16 ^A^	575.89 ± 507.79 ^B^	447.81 ± 415.44 ^C^	233.32 ± 324.18 ^D^
Lp (H)	−841.67 ± 1700 ^C^	−597.43 ± 1155.3 ^AB^	−642.08 ± 1219.2 ^B^	−508.65 ± 1213.8 ^A^
Ls (H)	−155.74 ± 231.36 ^B^	−127.86 ± 323.04 ^AB^	−127.79 ± 314.91 ^AB^	−101.73 ± 290.9 ^A^
R (kΩ)	715.97 ± 702.6 ^A^	508.86 ± 506.09 ^B^	394.65 ± 410.81 ^C^	204.6 ± 310.65 ^D^
X (kΩ)	−134.65 ± 174.08 ^B^	−112.78 ± 135.64 ^AB^	−115.95 ± 199.72 ^AB^	−95.41 ± 260.1 ^A^
DCR (kΩ)	1059.65 ± 928.51 ^A^	767.66 ± 672.26 ^B^	619.92 ± 553.36 ^C^	351.33 ± 430.64 ^D^
Z (kΩ)	763.3 ± 714.42 ^A^	542.55 ± 514.96 ^B^	420.84 ± 418.87 ^C^	218.28 ± 320.59 ^D^
ϴ (rad)	−0.28 ± 0.35 ^A^	−0.29 ± 0.3 ^A^	−0.38 ± 0.33 ^B^	−0.39 ± 0.3 ^B^
ϴ°	−15.87 ± 19.91 ^A^	−16.62 ± 17.04 ^A^	−21.79 ± 18.6 ^B^	−22.32 ± 16.86 ^B^
Q	0.48 ± 0.91 ^AB^	0.46 ± 0.69 ^B^	0.54 ± 0.65 ^A^	0.52 ± 0.53 ^AB^

The means (*n* = 720 for Lp parameter at 0, *n* = 979 for Lp parameter at 2-months, *n* = 980 for Lp parameter at 4 and 6-months, *n* = 1000 for all other parameters) within each column with the same letters are not significantly different at *p* ≤ 0.05.

**Table 5:** The correlation between the pH, total soluble solids (TSS), water activity (a_w_), and moisture content (MC) of the stored date fruits and the electrical parameters at various frequencies.

		Cs	Rs	D	Cp	Rp	Lp	Ls	R	X	DCR	Z	ϴ	ϴ°	Q
pH	10	−0.063	−0.120 **	0.375 **	−0.144 **	−0.140 **	00.054	0.307 **	−0.125 **	−0.087 *	−0.133 **	−0.123 **	0.423 **	0.427 **	−0.406 **
	100	−0.223 **	−0.082 *	0.722 **	0.461 **	−0.086 *	−0.222 **	00.062	−0.082 *	0.128 **	−0.126 **	−0.075 *	0.483 **	0.496 **	0.129 **
	1000	−0.280 **	−00.063	0.420 **	0.498 **	−0.077 *	−0.167 **	0.234 **	−00.062	00.047	−0.125 **	−00.067	0.306 **	0.350 **	−0.304 **
	10,000	−0.383 **	00.061	0.235 **	0.540 **	−00.042	−00.051	00.026	00.065	0.154 **	−0.120 **	−00.069	0.281 **	0.265 **	−0.223 **
	100,000	−0.454 **	0.325 **	0.176 **	0.559 **	0.111 **	0.027	0.030	0.374 **	−0.170 **	−0.069	0.237 **	0.059	0.076 *	−0.104 **
TSS	10	0.236 **	−0.307 **	−0.589 **	0.378 **	−0.302 **	−0.193 **	0.078 *	−0.322 **	−0.200 **	−0.305 **	−0.325 **	−0.621 **	−0.623 **	0.591 **
	100	0.231 **	−0.347 **	−0.189 **	0.292 **	−0.346 **	0.425 **	0.077 *	−0.349 **	−00.021	−0.321 **	−0.351 **	−0.433 **	−0.433 **	−0.220 **
	1000	0.395 **	−0.368 **	−0.153 **	0.229 **	−0.362 **	0.439 **	0.279 **	−0.369 **	00.011	−0.324 **	−0.366 **	−0.337 **	−0.360 **	0.339 **
	10,000	0.249 **	−0.410 **	−0.285 **	0.204 **	−0.366 **	0.250 **	0.120 **	−0.414 **	0.292 **	−0.328 **	−0.361 **	−0.435 **	−0.430 **	0.429 **
	100,000	0.618 **	−0.658 **	−0.116 **	0.172 **	−0.421 **	0.110 **	0.117 **	−0.665 **	0.444 **	−0.334 **	−0.542 **	−0.071 *	−0.080 *	0.007
MC	10	−0.160 **	−0.500 **	−0.293 **	0.102 **	−0.487 **	−0.225 **	0.270 **	−0.493 **	0.178 **	−0.465 **	−0.487 **	−0.504 **	−0.507 **	0.489 **
	100	−0.078 *	−0.492 **	−0.400 **	−00.028	−0.489 **	00.068	0.408 **	−0.488 **	0.402 **	−0.466 **	−0.491 **	−0.440 **	−0.434 **	−0.018
	1000	0.049	−0.497 **	−0.370 **	−00.038	−0.492 **	0.495 **	0.448 **	−0.501 **	0.434 **	−0.477 **	−0.500 **	−0.415 **	−0.446 **	0.413 **
	10,000	0.092 **	−0.492 **	−0.233 **	−00.056	−0.497 **	0.153 **	00.022	−0.497 **	0.484 **	−0.479 **	−0.511 **	−0.234 **	−0.224 **	0.244 **
	100,000	0.367 **	−0.366 **	−0.109 **	−0.080 *	−0.500 **	0.020	0.025	−0.395 **	0.469 **	−0.479 **	−0.475 **	0.120 **	0.115 **	−0.213 **
a_w_	10	0.186 **	−0.258 **	−0.644 **	0.268 **	−0.255 **	−0.159 **	−0.088 *	−0.265 **	−00.039	−0.244 **	−0.270 **	−0.643 **	−0.637 **	0.623 **
	100	0.164 **	−0.310 **	−0.403 **	−0.004	−0.309 **	0.070	0.071 *	−0.311 **	00.010	−0.254 **	−0.302 **	−0.574 **	−0.575 **	−0.153 **
	1000	0.292 **	−0.322 **	−0.290 **	−0.030	−0.319 **	0.487 **	0.168 **	−0.324 **	0.082 *	−0.249 **	−0.320 **	−0.524 **	−0.484 **	0.510 **
	10,000	0.299 **	−0.438 **	−0.224 **	−0.064	−0.350 **	0.324 **	0.174 **	−0.441 **	0.217 **	−0.252 **	−0.329 **	−0.378 **	−0.399 **	0.437 **
	100,000	0.610 **	−0.689 **	0.086 *	−0.096 **	−0.427 **	0.174 **	0.177 **	−0.616 **	0.513 **	−0.254 **	−0.568 **	0.076 *	0.089 *	−0.120 **

* The correlation is significant at the 0.05 level. ** The correlation is significant at the 0.01 level (2-tailed).

**Table 6:** A comparison between the ANNs models’ errors in the training, testing, and holdout phases at various frequencies.

Phases			Frequency (Hz)				
			10	100	1000	10,000	100,000
Training	Sum of squares error		130.82	135.43	101	96.1	102
	Average overall relative error		0.133	0.142	0.107	0.098	0.108
	Relative error	pH	0.122	0.120	0.079	0.058	0.075
		TSS	0.083	0.106	0.054	0.044	0.067
		MC	0.144	0.165	0.155	0.161	0.153
		a_w_	0.183	0.177	0.139	0.128	0.137
Testing	Sum of Squares Error		39.657	51.039	36.098	28.304	36.408
	Average overall relative error		0.140	0.172	0.121	0.098	0.119
	Relative error	pH	0.125	0.153	0.070	0.078	0.081
		TSS	0.084	0.143	0.075	0.053	0.084
		MC	0.165	0.206	0.184	0.143	0.161
		a_w_	0.188	0.182	0.146	0.113	0.158
Holdout	Average overall relative error		0.152	0.145	0.158	0.101	0.139
	Relative error	pH	0.160	0.163	0.120	0.082	0.105
		TSS	0.117	0.107	0.116	0.055	0.103
		MC	0.151	0.153	0.210	0.144	0.175
		a_w_	0.188	0.153	0.183	0.121	0.167

**Table 7:** Comparison between values of R^2^ and RMSE for the developed ANNs and MLR models in the evaluation phase at various frequencies.

Properties	Models	Frequency (Hz)									
		10		100		1000		10,000		100,000	
		R^2^	RMSE	R^2^	RMSE	R^2^	RMSE	R^2^	RMSE	R^2^	RMSE
pH	ANNs	0.878	0.14	0.871	0.191	0.924	0.122	0.938	0.121	0.924	0.129
	MLR	0.573	0.289	0.813	0.289	0.842	0.176	0.843	0.175	0.746	0.222
TSS (Brix)	ANNs	0.919	3.79	0.886	7.542	0.927	3.451	0.954	2.946	0.929	3.469
	MLR	0.734	6.215	0.609	6.215	0.563	7.975	0.735	6.208	0.76	5.903
a_w_	ANNs	0.816	0.034	0.823	0.039	0.862	0.026	0.876	0.02	0.859	0.024
	MLR	0.704	0.047	0.689	0.047	0.741	0.044	0.787	0.047	0.729	0.045
MC (%)	ANNs	0.853	0.852	0.826	1.239	0.841	0.816	0.855	0.803	0.844	0.816
	MLR	0.656	1.297	0.676	1.297	0.583	1.267	0.686	1.387	0.617	1.369

## Data Availability

Data is contained within the article.

## Nomenclature

ANNs	Artificial Neural Networks
a_w_	water activity
B	constant
C	capacitance, nF
Cp	capacitance value at parallel equivalent circuit model, nF
Cs	capacitance value at series equivalent circuit model, nF
D	dissipation factor
DCR	direct current resistance, kΩ
E_j_	error
L	inductance, H
Lp	inductance value at parallel equivalent circuit model, H
Ls	inductance value at series equivalent circuit model, H
MC	moisture content, %
M_j_	measured value
ML	Machine Learning
MLR	Multiple Linear Regression
n	number of measurements
O_j_	neuron output
pH	power of hydrogen
Q	quality factor
R	resistance, kΩ
R^2^	coefficient of determination
RMSE	root-mean-square error
Rp	equivalent parallel resistance, kΩ
Rs	equivalent series resistance, kΩ
t_j_	target value
TanH	hyperbolic tangent
T_i_	measured value
TSS	total soluble solids, Brix
W_j_	input weight
X	reactance, kΩ
X_i_	independent variable
X_oj_	the outcome of the sum value
y_i_	dependent variable
Z	the absolute value of the impedance, kΩ
θ	phase angle in radian, rad
θ°	phase angle, degree
